# Otosclerosis: Experience With Stapes Surgery

**DOI:** 10.7759/cureus.7927

**Published:** 2020-05-02

**Authors:** Muhammad Shaheryar Ahmed Rajput, Asif Ali Arain, Arsalan A Rajput, Mohammad Adeel, Anwar Suahil, Muhammad Sohail Awan

**Affiliations:** 1 Otolaryngology, Liaquat University of Medical and Health Sciences, Jamshoro, PAK; 2 Otolaryngology and Head and Neck Surgery, Aga Khan University Hospital, Karachi, PAK; 3 Otolaryngology and Head and Neck Surgery, King Faisal Specialist Hospital and Research Centre, Riyadh, SAU; 4 Otolaryngology, The Indus Hospital, Karachi, PAK; 5 Ophthalmology, Aga Khan University Hospital, Karachi, PAK; 6 Otolaryngology, Bradford Royal Infirmary, Bradford, GBR; 7 Otolaryngology, Shaukat Khanum Memorial Cancer Hospital and Research Centre, Lahore, PAK; 8 Head and Neck Oncology, Sheffield Teaching Hospitals National Health Service Foundation Trust, Sheffield, GBR; 9 Otolaryngology, Aga Khan University Hospital, Karachi, PAK

**Keywords:** otosclerosis, stapes, stapedectomy, stapedotomy, hearing loss

## Abstract

Introduction

Otosclerosis is a disorder in which the footplate of the stapes is replaced by an abnormal bone, thereby affecting sound transmission to the inner ear at the level of the oval window. The solution to this condition is to reestablish this mechanism back to normal via the ossicular chain to the inner ear. The aim of stapes surgery is to improve the hearing level to thresholds appropriate enough to obviate the need for hearing aid. The hearing improvement achieved after surgery often lasts for many years. The purpose of the current study was to review our experience and find out the rate of success related to hearing outcomes after stapedotomy.

Methods

The patients who were operated for otosclerosis between January 2000 and December 2010 at Aga Khan University Hospital, Karachi, Pakistan were included in the study. The charts were reviewed to collect clinical data regarding stapes surgery. The values of speech reception threshold (SRT) were recorded, and the preoperative and postoperative means were compared with a t-test. The bone conduction (BC) and air conduction (AC) thresholds were evaluated at 0.5 kHz, 1.0 kHz, 2.0 kHz, and 3.0 kHz. The preoperative and postoperative means of air-bone gap (AB-gap) were compared with a t-test. The descriptive frequency was calculated to evaluate postoperative AB-gap in individual patients; patients were grouped with a difference of 10 dB of AB-gap. The SPSS Statistics software (IBM, Armonk, NY) was used for statistical analysis.

Results

A total of 46 patients were included in the study. There were 15 males and 31 females. The mean age was 35 years (range: 20-56). Thirty-three patients had bilateral otosclerosis; two patients had surgery for both ears, taking the total number of ears operated to 48. The mean preoperative AB-gap was 39, while the mean postoperative AB-gap was 11. The means were compared with a t-test and a p-value of <0.05 was considered significant. The means of preoperative and postoperative SRT were 56.25 and 24.27 respectively. Both means were compared with a t-test, and a p-value of <0.05 was considred significant. Postoperatively, 34 ears had AB-gap of 10 dB (70.8%), 11 (22.9%) had within 20 dB, and three (6.3%) had within 30 dB.

Conclusions

The success rate related to hearing outcomes in patients operated for otosclerosis was excellent and comparable to that found in the current literature. The wide AB-gap noticed in the majority of our patients may represent a delayed presentation to otolaryngologists, which requires further evaluation.

## Introduction

Otosclerosis is an osseous disorder in which the footplate of the stapes is invaded and replaced by abnormal bone that causes interference with sound wave transmission to the inner ear at the level of the oval window. The solution to this condition is to reestablish the passage of sound waves back to normal via the ossicular chain to the inner ear. Otosclerosis is the most common cause of hearing loss in adulthood, with peak incidence occurring between the ages of 20-40. The disease affects 10% of the white population, and in 70-80 percent of the cases, both ears are involved [1,2]. The prevalence of otosclerosis is rare among Asians and the African population. One study has reported a higher prevalence of the condition in a community in South India, which is likely due to a higher incidence of marriages within the same community [3,4]. Pregnancy is known to aggravate the disease process; consequently, otosclerosis is more prevalent in females [5,6]. A familial history of hearing loss has been reported in 50% of the cases [7].

In 1741, ankylosis of the stapes was first described by Valsalva. Nearly one and half-century later, Toynbee described fixation of the stapes to the oval window. The evolution of modern surgery of otosclerosis has gone through distinct eras. At first, there was the mobilization of stapes, followed by the complete removal of stapes in the late 18th century, leaving the oval window open. The fenestration era began in the early 1900s when Holmgren performed surgeries to create fistula in the horizontal semicircular canal [2]. In 1955-56, Shea proved that the stapes could be removed and an oval window could be covered with a vein graft; Harry Treace made a biocompatible implant prosthesis out of the newly discovered Teflon that Shea had used to reconstruct the sound-conducting mechanism, and the era of stapedectomy was thus set in motion [8,9]. The technique of stapedectomy gained wide acceptance and has undergone various stages of improvement ever since. In the 1970s, Myers performed stapedotomy by the creation of a small hole in the footplate instead of complete or subtotal removal and used piston prosthesis. Perkins used laser for stapedotomy in the early 1980s [10]. However, a few challenges still remain with regard to the process, even though many believe that perfection has been achieved in the treatment of otosclerosis.

The aim of stapes surgery is to improve hearing levels to the thresholds appropriate enough to obviate the need for hearing aid. The hearing improvement achieved after surgery often lasts for many years [11]. The purpose of the current study was to review our experience and find out the rate of success related to hearing outcomes in patients who underwent stapes surgery for otosclerosis.

## Materials and methods

The database of Aga Khan University Hospital was searched to identify patients who were operated for otosclerosis between January 2000 and December 2010. The charts were reviewed to collect clinical data with regard to stapes surgery. The values of speech reception threshold (SRT) were recorded, and the preoperative and postoperative means were compared with a t-test. The bone conduction (BC) and air conduction (AC) thresholds were evaluated at 0.5 kHz, 1.0 kHz, 2.0 kHz, and 3.0 kHz. The preoperative and postoperative means of air-bone gap (AB-gap) were compared with a t-test. The descriptive frequency was calculated to evaluate postoperative AB-gap in individual patients; patients were grouped with a difference of 10 dB of AB-gap. Statistical analysis was performed by using the SPSS Statistics software (IBM, Armonk, NY).

## Results

A total of 46 patients were included in the study. There were 15 males and 31 females. The mean age was 35 years (range: 20-56). Thirty-three patients had bilateral otosclerosis; two patients had surgery for both ears, taking the total number of ears operated to 48. These variables are shown in Table 1.

**Table 1:** Age, gender, and clinical variables in patients

Variables		Values
Gender, n	Male	15
	Female	31
Age, years	Mean	35
	Range	20-56
Laterality, n	Unilateral	13
	Bilateral	33
Surgery, n	Both ears operated	2
	One ear operated	44

The mean preoperative AB-gap was 39, and the mean postoperative AB-gap was 11. The means were compared with a t-test, and the p-value was found to be significant. A comparison of the means of AB-gap is shown in Table 2.

**Table 2:** Comparision of preoperative and postoperative AB-gap with a t-test AB-gap: air-bone gap

AB-gap	Mean	Standard deviation	T-test
Preoperative	39	5.9	P-value: <0.001
Postoperative	11	4.2	

The means of preoperative and postoperative SRT were 56.25 and 24.27 respectively. Both means were compared with a t-test, and the p-value was found to be significant, as shown in Table 3.

**Table 3:** Comparison of preoperative and postoperative SRT with a t-test SRT: speech reception threshold

SRT	Mean	Standard deviation	T-test
Preoperative	56.25	8.6	P-value: <0.001
Postoperative	24.27	5.8	

Postoperatively, 34 ears had an AB-gap of 10 dB (70.8%), 11 (22.9%) had within 20 dB, and three (6.3%) had within 30 dB. The details of the postoperative AB-gap are shown in Table 4.

**Table 4:** Postoperative hearing benefit based on AB-gap closure AB-gap: air-bone gap

AB-gap closure	Number of ears	Percentage
<10 dB	34	71
>10 to <20 dB	11	23
>20 to <30 dB	3	06

## Discussion

In 2013, a report on 262 stapedotomies performed on 228 patients showed closure of AB-gap to less than 10 dB in 86% of cases and close to 20 dB in 11% of cases [12]. Another study in 2015 showed similar results with 87% of the patients having residual AB-gap of less than 10 dB. However, two of their patients had complete sensorineural hearing loss. The first patient had labyrinthitis in the immediate postoperative period and developed a deaf ear by the 10th postoperative day. The second patient presented with sudden deafness after three months of surgery [13]. In the current study, comparable results have been seen with 71% of the patients achieving AB-gap within the range of 10 dB. The values of the SRT have shown excellent improvement on postoperative hearing assessment. The majority of the patients in the current study were found to have a preoperative AB-gap close to 40 dB. In 2019, a study by Deniz et al. analyzed the factors affecting the postoperative outcome in patients with otosclerosis. The authors found that a large AB-gap in patients with otosclerosis was a poor prognostic indicator for the postoperative hearing outcome. They proposed an AB-gap cut-off value of <34.5 dB as a possible parameter to predict outcomes in clinical practice [14].

The microscope is the conventional method used for middle ear surgeries, and the endoscope has been recently utilized as an alternative technique to view middle ear structures [15,16]. A microscope allows binocular vision and freedom of both hands with an unobstructed direct view of the operating field [17,18]. Now the endoscopes have become the modern way of visualization in the surgical domain [19]. Recent studies on endoscopic stapes surgeries and outcomes have reported excellent audiological success rates [15,18]. All patients in the current study underwent conventional microscopic middle ear surgery.

Sensorineural hearing loss and vestibular dysfunction are the major complications encountered after stapes surgery. These complications are thought to be caused by mechanical trauma resulting from the use of a micro drill or thermal trauma when a laser is used to create a hole in the stapes footplate. For this reason, the perioperative administration of corticosteroid has recently been initiated. Szekely et al. have reported the efficacy of perioperative use of intravenous corticosteroid treatment. The authors concluded that it could be a subject of further investigation in a prospective manner [20]. None of the patients in the current study had such complications.

Carhart notch was first described in 1950. It involves a downward deflection in the bone curve at a frequency of 2,000 Hz on a pure tone audiogram by approximately 10-20 dB. It possibly arises as a result of mechanical fixation of the stapes base in the oval window that causes disturbances in the self-resonance. A study that considered the Carhart notch as one of the factors affecting the postoperative hearing has concluded that stapedotomy is the procedure with the highest efficiency in the treatment of otosclerosis regardless of the presence or absence of the Carhart notch [21].

## Conclusions

Despite the wide preoperative AB-gap in the majority of the patients, the results of the study revealed excellent outcomes with stapes surgery for otosclerosis. These results further reinforce the understanding that the treatment of otosclerosis with stapes surgery is highly efficient. The large number of AB-gaps may be attributed to a delayed presentation or a societal attitude of not following the advice to undergo early surgery. However, this phenomenon requires further studies so that a clear understanding can be achieved.
